# A Narrative Review on Pediatric Scurvy: The Last Twenty Years

**DOI:** 10.3390/nu14030684

**Published:** 2022-02-06

**Authors:** Sandra Trapani, Chiara Rubino, Giuseppe Indolfi, Paolo Lionetti

**Affiliations:** 1 Pediatric Unit, Department of Health Sciences, Meyer Children’s University Hospital, University of Florence, Viale Pieraccini 24, 50137 Florence, Italy; 2 Pediatric Unit, Meyer Children’s University Hospital, Viale Pieraccini 24, 50137 Florence, Italy; chiara.rubino@meyer.it; 3 Pediatric Unit, Department of NEUROFARBA, Meyer Children’s University Hospital, University of Florence, Viale Pieraccini 24, 50137 Florence, Italy; giuseppe.indolfi@unifi.it; 4 Gastroenterology Unit, Department of NEUROFARBA, Meyer Children’s University Hospital, University of Florence, Viale Pieraccini 24, 50137 Florence, Italy; paolo.lionetti@unifi.it

**Keywords:** scurvy, children, musculoskeletal pain, limp, hemorrhage, ascorbic acid

## Abstract

Scurvy is a well-known clinical condition caused by vitamin C deficiency. Although considered a rare disease in high-income countries, it has been recently increasingly reported in children, especially in those with abnormal dietary habits, mental or physical disabilities. We performed an extensive review of the literature analyzing studies published in the last 20 years focusing on clinical features, differential diagnosis and diagnostic delay. Fifteen articles were selected, collectively reporting a total of 166 children. Because of the wide clinical spectrum (musculoskeletal complaints and/or mucocutaneous lesions or systemic symptoms), scurvy can mimic several conditions, including autoimmune diseases, infections, and neoplasia. In addition, frequent findings such as normal nutritional status, anemia or elevated inflammatory markers may guide clinicians towards the abovementioned misdiagnoses. Scurvy should be considered in patients presenting with musculoskeletal complaints, not only in those with risk factors but also in healthy children. A focused dietary history and a careful physical examination, assessing other signs of vitamin C deficiency, are mandatory in these patients. When suspected, the dosage of serum vitamin C is the diagnostic gold standard; furthermore, imaging studies, performed by an expert radiologist, can reveal the typical features of scurvy. Only early diagnosis can avoid unnecessary investigations and potentially fatal complications of the disease.

## 1. Introduction

Scurvy is a well-known clinical syndrome that results from vitamin C deficiency [1]. Being rare as compared to other nutritional deficiencies in childhood, it is rarely suspected, and this frequently leads to a delayed diagnosis [2,3]. Moreover, the disease spectrum of scurvy is quite variable, including musculoskeletal, dermatological, dental and systemic manifestations, and this aspect can contribute to misdiagnosis. In the last years, scurvy is re-emerging in the literature, especially in children with abnormal dietary habits, mental illness, chronic renal diseases or physical disabilities [2,4,5,6]. Updated data on its prevalence in European children are not available. Based on the 2003–2004 cross-sectional study by the National Health and Nutritional Examination Survey in the United States, the prevalence of vitamin C deficiency in the total population, which included subjects aged more than 6 years, was about 7.1%, though the majority were clinically asymptomatic [7].

When vitamin C values become lower than 0.2 mg/dL and the total body value falls below 300 mg, the clinical manifestations of scurvy appear [8]. A balanced diet could be sufficient to prevent scurvy, but sometimes this does not occur, as in healthy children presenting food aversion. Globally recommended intake for children and adolescents, lower than adults, are generally based on their body weight [9]. In well-nourished populations, such as in the United States, the vitamin C level in children is higher (mean 71 mol/L) and the prevalence of its deficiency is lower than in adults. In the National Health and Nutritional Examination Survey 2003–2004, when the various age groups are analyzed, the prevalence of vitamin C deficiency was 1.6% in children aged 6–11 years and <4% in adolescents [7]. However, this does not occur in low and middle-income countries where low vitamin C status has been frequently observed in youngsters. Similarly, studies in Mexico have shown that vitamin C concentrations in school-aged children were low, with up to 38% hypovitaminosis C, and 23% deficiency [10,11].

Although its prevalence in childhood is not precisely known in high income countries, certain pediatric populations are at higher risk, specifically children with mental, neurological or physical disabilities, autism [4,12,13,14], eating disorders, patients on restrictive diets, end-stage renal disease patients on dialysis [15], patients with gastrointestinal malabsorption [16], iron overload from multiple transfusions related to sickle cell anemia [3] and patients with inadequate enteral feedings as the sole source of nutrition [17]. However, healthy children may also be affected, surprisingly [18,19,20]. Its early diagnosis and appropriate treatment generally have gratifying results, but untreated scurvy can be fatal, with deaths reported from infection, cerebral hemorrhage [21] or hemopericardium [22,23]. In the present narrative review, we summarized the fundamental role and the pathophysiology of vitamin C in the human body and the source of this nutrient. From the literature, we analyzed all pediatric case series of scurvy in the last twenty years, focusing on clinical features, comorbidities, laboratory alterations, radiological features, treatment and outcome. Moreover, the differential diagnosis with various severe diseases and the delay to reach such a diagnosis have been stressed.

### 1.1. Role of Vitamin C

**Figure 1:**
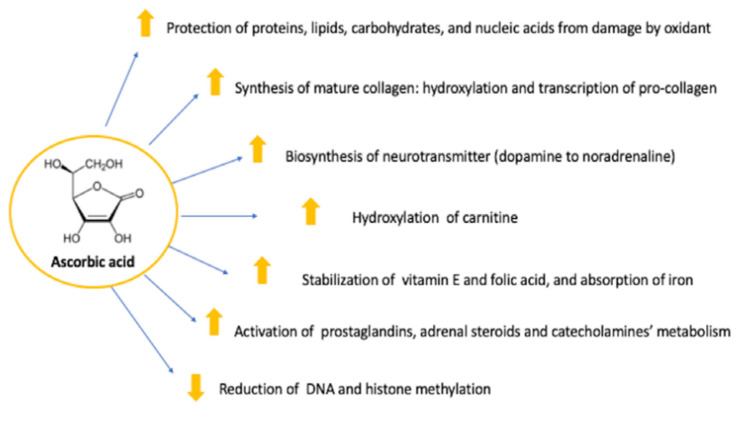
Roles of ascorbic acid in several biochemical reactions.

Vitamin C (L-ascorbic acid or ascorbate) is an essential water-soluble micronutrient for humans who cannot synthesize it due to the loss of a key enzyme in the biosynthetic pathway [24,25]. The enzymatic process for conversion of glucose to ascorbic acid via gluconolactone oxidase is not possible for humans, therefore vitamin C intake in the form of fresh fruits, vegetables or dietary supplements is essential [26]. Other animals devoid of the ability of endogenous vitamin C synthesis include nonhuman primates, guinea pigs, Indian fruit bats and several bulbul varieties [26]. This nutrient plays various important roles as a cofactor, enzyme complement, co-substrate, reducing agent and an antioxidant in several biochemical reactions (Figure 1). The reduced form of this vitamin, ascorbic acid, is an especially effective antioxidant due to its ability to readily donate electrons, thus protecting important biomolecules (proteins, lipids, carbohydrates and nucleic acids) from damage by oxidants generated during cell metabolism and through exposure to toxins and pollutants [27]. Moreover, it is an enzymatic cofactor necessary for the synthesis of mature collagen, formed by three polypeptide molecules combined into a triple helix. Collagen type IV is the main constituent of blood vessel walls, skin and, specifically, the basement membrane zone separating epidermis from dermis. Vitamin C allows hydroxylation and transcription of pro-collagen; thus, a lack of this nutrient induces collagen abnormalities, which explains the main clinical manifestation of scurvy. Furthermore, it stabilizes several other compounds, including vitamin E and folic acid, and enhances iron absorption, reducing it to a more absorbable ferrous state. It may regulate inflammatory response by playing a role in the metabolism of prostaglandins, adrenal steroids and catecholamines. In the last decades, the high contribution of vitamin C to immune defense has been stressed by supporting various cellular functions of both the innate and adaptive immune systems [28]. Overall, based on its newly discovered epigenetic roles, vitamin C may be essential for the growth and development of infants and children [29].

Vitamin C deficiency induces collagen abnormalities that explain some clinical manifestations of scurvy: abnormal dentine production and loss of teeth, vessel wall damage and bleeding, purpura, edema, bone changes (in children) due to the inability of osteoblasts to produce the osteoid seam and skin changes related to keratin abnormalities [30].

Additionally, it is involved in the synthesis of tyrosine, norepinephrine, epinephrine and carnitine and the metabolism of cyclic nucleotides in humans. Carnitine, requiring vitamin C for its hydroxylation, is an essential cofactor in the transport of long-chain fatty acids into the mitochondrial matrix. Carnitine deficiency may be responsible for the early symptoms of scurvy, such as lack of energy and muscle aching. Vitamin C is involved in neurotransmitter biosynthesis (hydroxylation of dopamine to noradrenaline) and the amidation of neuroendocrinal hormones such as gastrin, bombesin, corticotropin-releasing hormone and thyrotropin-releasing hormone. Such altered biosynthesis of catecholamines may explain the behavior and mood disorders associated with vitamin C deficiency.

### 1.2. Metabolism, Dietary Needs and Sources of Vitamin

The total pool of Vitamin C in the body is 1500 mg, corresponding to 2 mg/100 g body weight for a 75 kg individual, and the daily turnover is 45–60 mg (about 3% of the total) [9]. Estimates of body pool and turnover have been based on studies using ^14^C-labelled ascorbate performed in the 1960s and 1970s [31,32,33]. Although several aspects of vitamin C pharmacokinetics were not known at that time, thereafter these estimates have been performed in human modeling studies with similar results [34]. Due to such little storage, plasma concentration of vitamin C is related to the recent intake and the half-life of ingested vitamin C (ranging from 10 to 20 days) depends on the individual vitamin C status [35]. As it is water-soluble, vitamin C is well absorbed: its uptake starts with its passage through the mouth and absorption by the oral mucosa but mainly takes place in the jejunum and ileum; from the gastrointestinal tract, it is located in both intracellular and extracellular tissues. The absorption rate is about 85%, and it occurs mainly via an active sodium-ascorbate cotransport mechanism in enterocytes. It is likely because of the limited capacity of this cotransport that large oral doses of ascorbate are absorbed less completely than small doses [8,36]. Other mechanisms of intestinal absorption are passive or facilitated diffusion. Therefore, vitamin C plasma concentration is exclusively related to the daily intake. Vitamin C is subsequently distributed to the organs mainly through active transport [35]. Several organs, particularly the brain, have concentration-dependent mechanisms for vitamin C retention, thereby maintaining adequate levels in case of inadequate intake at the expense of other organs. Ascorbic acid is filtered through the renal glomeruli and concentrated after the reabsorption of water. Reuptake of ascorbic acid in the proximal renal tubules occurs through saturable active transport [35]. Renal reabsorption and excretion contribute to tight control of plasma vitamin C concentrations [37]. Erythrocytes play an important role in vitamin C homeostasis. Through facilitated diffusion, erythrocytes maintain an intracellular vitamin C concentration similar to that of the plasma. Inside the erythrocytes, the oxidation product of ascorbate is reduced to ascorbate, which is then released in plasma. This mechanism of recycling depends on reduced glutathione and represents an antioxidant reserve [35,38,39]. The international recommended daily allowances (RDAs) for vitamin C are variable. For example, the World Health Organization recommends a daily vitamin C intake of 45 mg with the objective of preventing vitamin C deficiency [40]. On the other hand, other European countries, such as Italy, based their RDAs on “an amount of vitamin C that is thought to provide antioxidant protection as derived from correlation of such protection with neutrophil ascorbate concentrations” resulting in RDAs of 75–100 mg/day [9]. Notably, some studies reporting the combined evidence from human metabolic, pharmacokinetic and observational studies and Phase II randomized controlled trials suggested that 200 mg is the optimum daily vitamin C intake of vitamin C for the majority of the adult population to maximize the vitamin’s potential benefits with the least risk of inadequacy or adverse effects [41]. Pediatric RDAs for vitamin C are generally derived from adult needs and adjusted to their lower body mass [9,42]. The RDA of vitamin C is 15–45 mg for age 1–13 years and 65–75 mg for age 14–18 years. In Italy, for example, the Italian Society for Human Nutrition recommends an intake of 45 mg for children from 4 to 6 years of age [43]. The requirement for vitamin C increases during infections, inflammatory states, pregnancy, and lactation. The vitamin C level in the newborn is related to the maternal levels as the vitamin is transported by active placental transfer and subsequently maintained by secretion of vitamin C in breast milk [2]. Breast milk and fortified formula are richer in vitamin C than cow’s milk; therefore, the risk of scurvy occurrence is low in the first year of life since, during their first six months, infants have mostly breast or formula milk, both rich in Vitamin C (more than cow’s milk). During the first phase of weaning, fruits and vegetables that contain vitamin C are usually included in daily nourishment. Up to 90% of Vitamin C is taken in the form of vegetables and fruits. The best sources of vitamin C are citrus fruits (oranges, lemons, tangerines, limes, grapefruits, kiwis, black currents, melons) and vegetables (tomato, potato, cabbage, broccoli, spinach, lettuce, cucumber, red pepper). However, many of these foods can lose their vitamin C content because of cooking, storage or oxidation [28].

## 2. Materials and Methods

We performed an extensive literature review of all reported pediatric cases of scurvy. Such a review was conducted using Embase^®^, MEDLINE^®^, MEDLINE^®^-In Process to identify studies on scurvy in childhood published as full-text articles from January 2000 to November 2021. Databases were searched combining the keywords “Scurvy” or “Vitamin C deficiency” or “acid ascorbic deficiency” AND “child” OR “children” OR “infancy”. We included the articles which matched the following eligibility criteria: (1) they provided original data on case series including more than two patients affected with scurvy; the series were selected as more representative than the single case reports which have not been included; (2) the patients reported were younger than 18 years; (3) they were written in English.

## 3. Results

Applying our search strategy, we found 15 articles describing three or more children with scurvy. The majority (11/15, 73%) had been published after 2015 and only 4 (27%) were published between 2000 and 2014. The number of reported patients in the selected publications ranged between 3 and 48 children; collectively, the studies included a total of 166 patients. Their comorbidities, clinical, laboratory and radiological findings are summarized in Table 1. Details about single studies are reported in Table S1.

### 3.1. Epidemiology

Data about age and gender were reported in 13/15 studies corresponding to 118 patients. Their median age was 42 months (interquartile range—IQR 29-96), ranging from 5 to 132 months. Consistently, with regards to gender distribution, a high preponderance of males (87/118, 74%) was noted. 

### 3.2. Underlying Diseases/Risk Factors

The majority of patients (127/166, 76%) had a comorbidity associated with an increased risk of vitamin C deficiency. The most common comorbidities were neurological conditions such as autism, anorexia, cerebral palsy and developmental delay, altogether described in 29%. In second place, hematological disorders, including transfusion-related iron overload, bone marrow transplant recipients and chronic graft versus host disease, were found in 14%. It is worth noting that more than a quarter of cases (29%) showed severe malnutrition as a concomitant condition. Only 39 patients (23%) were otherwise healthy. 

### 3.3. Clinical Manifestations

Clinical manifestations were assessed in 13 studies and 86 children. The earliest manifestations are nonspecific, including constitutional symptoms such as fever (17%), malaise and asthenia (13%), irritability (10%) and loss of appetite (7%). Notably, 80% of patients had poor nutritional status. Musculoskeletal complaints were the most frequently described manifestations (92%); particularly, severe pain in the lower limbs (88%) with refusal to walk (73%) and limping (31%). These manifestations are present in a high but variable rate of patients with scurvy, ranging from 67% [44] to 100% [18,45,46] in the different series. Rarely, pain in the back (1%) or the upper limbs (1%) was referred, too. Mucosal involvement was reported in 57% of patients, most frequently with gingival bleeding (43%) and hypertrophy (27%) or, seldom, epistaxis (5%). The cutaneous lesions included petechiae or ecchymoses (39%), perifollicular hemorrhage (7%), hyperkeratosis presenting as dry rough skin (2%) and corkscrew hairs (3%). Poor wound healing, ulcers, worsening of pre-existing acne and subcutaneous nodules are occasionally described. Other unusual manifestations included pulmonary hypertension (3%) and abdominal pain (2%).

### 3.4. Diagnostic Delay and Provisional Diagnosis

Difficulty in diagnostic assessment and the differential diagnosis was underlined by several authors: 11 studies reported the interval between clinical onset and diagnosis, ranging from one week [47] to 2 years [48]; when available, the median interval was 2 months, calculated on 9 studies [4,5,18,45,46,49,50,51,52]. Thirty patients (18%) underwent invasive diagnostic exams: 11 bone marrow aspiration/biopsy, five bone biopsy, three gingival biopsies, two bone scintigraphy, two head CT scans, two chest-abdomen CT scans, two brain MRIs, one muscle biopsy and one lumbar puncture.

In a consistent percentage of patients (32%), one or more provisional misdiagnoses were made before reaching the correct diagnosis of scurvy. Oncologic conditions, as leukemia and bone neoplasia were hypothesized in 25 and 5 cases, respectively (52% and 10%). Secondly, infectious diseases of the musculoskeletal system, including osteomyelitis/septic arthritis (29%), myositis (6%) or spondylodiscitis (2%) were suspected. Chronic nonbacterial osteomyelitis (CNO) and juvenile idiopathic arthritis (JIA) were firstly diagnosed in 8 (17%) and 3 cases (6 %), respectively.

### 3.5. Laboratory Findings

Inflammatory markers were evaluated in 7 studies [4,46,47,48,50,51,52], assessing a total number of 42 patients, and were found to be elevated in 25 (59%). In particular, erythrocyte sedimentation rate (ESR) was elevated in 15 cases and C-reactive protein (CRP) in 13 cases. Anemia is another hallmark of scurvy, and it may be secondary to a combination of bleeding and decreased iron absorption or abnormal folate metabolism. It was assessed in 83 cases in 12 studies [4,5,18,44,45,46,47,48,49,51,52,53] and present in 46% of children. Complete nutritional blood tests should be performed and may reveal further multiple and severe vitamins deficiencies. Eight studies [4,18,46,47,48,49,50,51] reported other associated vitamin deficiencies in 45 patients, overall. The most frequently found was vitamin D deficiency (60%), followed by vitamin B1, B6 and A (9%, each one) deficiencies; other vitamins or minerals deficiencies were reported in lower percentages. Notably, the exact value of vitamin C was reported in 44 cases and its median value was 0.1 mg/dL.

### 3.6. Radiological Findings 

A plain X-ray was assessed in 125 patients. Typical alterations were present in most cases. Classic scurvy X-ray signs referred to in most studies were calcification at the metaphysis (lines of Fränkel, 73%), calcification around the epiphysis (Wimberger ring sign, 41%), osteopenia (31%), metaphyseal spurs (Pelkan spurs, 18%) and a “scorbutic zone” of a lucent metaphyseal band (Trümmerfeld zone, 14%). All these alterations were variably combined. Notably, the typical scurvy alterations were remarked only after a review following the diagnosis of scurvy in 8 children [4,47,51]; only in 7% of cases X-ray did not show any alterations. Magnetic Resonance Imaging (MRI) was assessed in 36 cases in 11 studies [3,4,18,45,46,47,48,49,50,51,52] and alterations consistent with scurvy were found in 97% of cases. The MRI findings in scurvy reflect the underlying pathophysiology, with areas of hemorrhage seen within bones and in the periosteum; increased periosteal reactions were described in 5 cases. Furthermore, multifocal symmetrical signal abnormalities involving the metaphysis with associated marrow enhancements were reported in 17 patients (47%); abnormal confluent T1 hypointense/T2 hyperintense signals in multiple bones are usually present [54]. Computed Tomography (CT) is not usually performed as it provides limited information about bone marrow; from the literature review it was done only in 6 cases, and was abnormal in 5: bone rarefaction/reabsorption and focal contrast uptake were the alterations described [4,46,52]. 

### 3.7. Management and Outcome 

Treatment and outcome were reported in 76 cases: only in 10 (13%) patients was vitamin C administered first intravenously and then orally; in the remaining 66 (87%), it was administered orally for the entire treatment course. The doses reported in infants and children ranged from 100–300 mg to 4000 mg daily [48], in divided doses. Treatment length varied from 2 weeks to 7 months with a mean duration approximately of 1 month or until full recovery of clinical signs and symptoms occurs. Following treatment initiation, a complete recovery of clinical manifestations was described in 58/76 (76%) cases, after an interval ranging from 1 to 11 months. A partial symptom improvement was reported in 14 children (18%), whereas 4 cases (5%) were lost at follow-up.

## 4. Discussion

Although considered a rare disease in high income countries, scurvy has been recently increasingly reported in children [55]. In literature, the large majority of children with scurvy are reported to have underlying conditions leading to inadequate nutrition such as neuropsychiatric [14,56,57] or neurological and developmental disorders [3,58]. Some other diseases can also reduce the absorption of vitamin C (ulcerative colitis, Crohn, Whipple’s and celiac disease) and/or increase the amount needed by the body (hemodialysis, bone marrow transplant, and chemotherapy) [3]. Low vitamin C concentrations can also occur in patients with iron overload (multiple blood transfusions in sickle cell anemia or thalassemia) which leads to wasting of vitamin C by the kidneys [3,59]. However, recent case reports and case series described scurvy also in healthy children without known risk factors; these children, called “picky eaters”, choose to take very restrictive and selective diets mainly based on carbohydrates [20,45,60,61]. In the large majority of these cases the consumption of vegetables and fruit is not present. Although causes of picky eating have not been definitively established, they appear to include early feeding difficulties, late introduction of lumpy foods during weaning, parents’ pressure to eat and early child choosiness, especially if the parents are worried about it [62]. Clinical manifestations usually develop after 8–12 weeks of inadequate vitamin C intake [2]. The initial manifestations are non-specific such as asthenia, irritability, loss of appetite and low-grade fever. Cutaneous manifestations, such as petechiae, ecchymoses, and hyperkeratosis can develop later, after about five months of a vitamin C-deficient diet. Occasionally, the purpura becomes palpable and the ecchymoses widespread, thus mimicking vasculitis [63]. Some patients present alopecia or develop hair fragility; a particular hair growth called “corkscrew hairs” is also observed, although rarely. Gums become swollen, loosen and bleed on slight pressure in a majority of patients. Musculoskeletal manifestations such as limping, arthralgia, myalgia, and limb or joint swellings generally follows other signs, but in childhood they may represent the first clinical manifestation requiring medical attention, with or without cutaneous manifestations [8,50,64,65]. Lower extremities are commonly affected, especially ankles, knees and hips; however, any joint can be involved. Leg and hip pain i the main reason for difficulty or refusal of bearing weight or walking and, in many cases, is associated with edema. In infancy, scurvy may have features of pseudo-paralysis, simulating osteomyelitis or septic arthritis. A true synovitis of affected joints is rare, while spontaneous hemarthrosis is common [8,63]. Other rare and potentially lethal manifestations include pulmonary hypertension, diminished adrenal and bone marrow function, poor wound healing, and severe anemia which can lead to cardiac hypertrophy up to high output heart failure [2,66]. As mentioned above, scurvy has a varied spectrum of clinical features and the presence of isolated symptoms may be confusing. Moreover, it can mimic many common diseases such as septic arthritis, osteomyelitis, abscess, or malignancies such as leukemia or bone neoplasia, thus, the disease can be belatedly diagnosed. Another misleading factor is the adequate growth with normal body mass index in a consistent proportion of patients [4,46,48]. Laboratory investigations typically show increase in inflammatory markers, especially ESR [47,50,51]. In addition, anemia was found in a high percentage of children with scurvy, and it is due to multiple causes. It can be microcytic if associated with iron deficiency, more rarely normocytic when secondary to bleeding, intravascular hemolysis or chronic underlying conditions or even macrocytic when combined with other dietary deficiencies such as vitamin B12 and folic acid [2,65,67,68]. Both elevated ESR and anemia may represent a confounding factor, as they are commonly also increased in immune-mediated conditions, infectious diseases and malignancies. Leukopenia and hypoalbuminemia may also be found as markers of malnutrition. Concomitant micronutrient deficiencies other than vitamin C deficiency may accompany scurvy; therefore, the clinicians should also check for levels of zinc, iron, folate and the vitamin B group [69]. A concurrent vitamin D deficiency, associated with incorrect nutrition, is reported in several series [18] and case reports [12,22]. Measurement of serum vitamin C is the gold standard for diagnosis, and serum levels below 200 mcg/dL are considered deficient; however, it may be adequate in the case of recent ascorbic acid intake [2]. Therefore, the most relevant diagnostic clue is the clinical resolution after ascorbic acid treatment, which occurs within 1 week to 3 months. The typical radiographic findings usually appear after 3–6 months of vitamin C deficiency; thus, most patients in the early stage of the disease have normal radiographs or findings of diffuse demineralization. Subsequently, classic findings develop at the distal ends of the long bones and are usually present at the knees and ankles; however, the pelvis and sacroiliac bone can also be also affected. The most specific radiographic features include Fränkel’s line, an irregular but thickened white line in the metaphyseal endings, representing the zone of well-calcified cartilage and an adjacent Trummerfeld zone, a rarefaction secondary to poorly formed trabeculae [55,70]. In addition, the Pelkan spur represents a healing metaphyseal pathological fracture and the Wimberger ring sign denotes a thin sclerotic rim surrounding a small lucent epiphysis [2,3]. Typical MRI findings include the hyperintense T2-W signal in the bone marrow (edema-like) of the metaphysis, increased periosteal reaction and significant soft-tissue edema adjacent to the involved skeletal segments [3,47]. Imaging findings in scurvy, especially at disease onset, could not be as evident as expected, especially for fledging radiologists. The abnormal bone marrow MRI appearance and subperiosteal hemorrhages are early and key features of scurvy. When abnormal bone marrow is present around the joint, radiologists should consider expanding field of view imaging in order to evaluate for symmetry and multifocal lesions [54]. CT provides limited information regarding the characterization of bone lesions and bone marrow; therefore, it should be reserved for those cases who require drainage or surgical intervention. There are no standardized treatment regimens: in the pediatric age group, the dose ranges from 100–300 mg daily to 1000 mg daily for 1 month or until full recovery [48]. Divided doses distributed throughout the day should be used as intestinal absorption (and renal excretion) mechanisms become saturated with intake greater than 100 mg [8]. Parenteral administration is required only in patients with malabsorption. The spontaneous bleeding, oral and constitutional symptoms generally improve at first, stopping within a few days; later, the bone abnormalities and ecchymoses gradually resolve over several weeks; a complete resolution of other clinical findings, including hematologic disorders, usually occur by 3 months [5]. As already mentioned, diagnosis of scurvy can be challenging because clinical and laboratory findings can mimic several conditions such as oncological disorders (leukemia, lymphoma, osteosarcoma or metastatic neuroblastoma), musculoskeletal infectious (septic arthritis, osteomyelitis, pyomyositis, CNO) [68], and rheumatic diseases (vasculitis such as Henoch Schoenlein purpura, and JIA) [71,72,73]. These diagnostic hypotheses were made even in cases presenting with associated malnutrition, demonstrating clinicians’ low awareness of scurvy as it is considered a “forgotten disease” in Western countries. Imaging studies can help to guide differential diagnosis, but they must be supplemented with a focused dietary history, a thorough physical examination, and a few laboratory tests. A detailed dietary anamnesis with the parents can reveal the selective diet that was initially not highlighted. Whenever not readily diagnosed, the outcome may be unfavorable; the consequences of scurvy may be relevant and include intensive and invasive investigations, hospital admission with prolonged hospital stays, physical disability and even severe complications such as severe infections and cerebral hemorrhage or hemopericardium, which may be fatal if also untreated in children [74,75].

## 5. Conclusions

Scurvy is one of the oldest known diseases. In the past, scurvy selectively affected homeless, poor and elderly subjects. Furthermore, it is well known that several groups of diseases (neurological, psychiatric, renal, and malabsorptive conditions) predispose to develop ascorbic acid deficiency. In the last decades, reports of scurvy, even in apparently healthy children, are more and more frequent. Even now, scurvy, despite being an uncommon condition, represents a potentially fatal disease. Pediatricians should consider scurvy in all children presenting with musculoskeletal complaints, especially in patients with risk factors for a selective diet. It can present itself in various forms, mimicking the presentation of several common diseases (infectious and autoimmune diseases, hematological and solid malignancies). Thus, differential diagnosis is complicated and can be even more challenging because children may have a normal nutritional and developmental status and laboratory tests can reveal anemia or elevated inflammatory markers, which are common findings in inflammatory, infectious and oncological diseases. Therefore, a high index of suspicion is required for a pediatrician, who should search for other signs of vitamin C deficiency at physical examination such as gingival alterations or ecchymosis. A detailed dietary history is mandatory in order to raise the possibility of scurvy, and should always be assessed, preferably at the first evaluation or during the disease course. In the presence of undefined condition, imaging studies such as X-Ray and MRI can reveal the typical features of scurvy if done by expert radiologists. In conclusion, scurvy can be a challenging diagnosis that frequently takes a long time to be reached. However, when focusing on a careful assessment of the child’s dietary habits, together with the evaluation of comparative bilateral X-ray or MRI, diagnosis of scurvy can be easily made avoiding unnecessary investigations such as extensive laboratory workup and bone biopsy. The presence of gingival ulceration combined with the refusal to walk and lower extremity myalgias/arthralgias with edema are highly suggestive of vitamin C deficiency. The increasing number of healthy children with scurvy highlights the necessity for clinicians to consider qualitative nutritional disorders as seriously as quantitative ones, widely described in adolescents (anorexia, bulimia). Pediatricians from developed countries are already managing the well-known “junk food” issue: often linked to socio-economic instability and limited access to healthy foods, junk food may be associated not only with obesity and metabolic syndrome but also with nutritional imbalance, especially if not fortified. In later years, pediatricians are also facing the emergence of various restrictive and bizarre diets. Avoidance of certain nutrients because of child tantrums or presumed allergies has brought back scurvy in developed countries and should be discouraged by pediatricians.

## Figures and Tables

**Table 1:** Clinical, laboratory and radiological features of the selected 15 studies describing 166 children with scurvy.

Underlying Diseases Assessed in 166		Clinical Manifestations Assessed in 86		Laboratory Data		Radiological Findings		Provisional Misdiagnosis Assessed in 48	
**Neurological** Autism Cerebral Palsy Neurological Delay Other **Hematological** Iron Overload Chemotherapy **Other** Severe Malnutrition Hereditary Fructose Intolerance Infantile Tremors Neglect	48(29%) 26(16%) 6 (4%) 6 (4%) 9 (5%) 23(14%) 20(12%) 2 (2%) 48(29%) 4 (2%) 3 (2%) 1 (1%)	**Musculoskeletal** Lower Limb Pain Refusal To Walk Arthritis/Limb Edema Limping Upper Limbs Pain Back Pain **Mucosal** Bleeding Gums Hypertrophy Gums Epistaxis Conjunctival Bleeding **Cutaneous** Petechiae/Ecchymosis Perifollicular Hemorrhage Corkscrew Hairs Hyperkeratosis **Others** Poor Nutritional Status Fever Malaise/Asthenia Loss of appetite Irritability Pulmonary Hypertension Abdominal Pain Photophobia	79 (92%) 76 (88%) 63 (73%) 28 (33%) 27 (31%) 1 (1%) 1 (1%) 49 (57%) 37 (43%) 23 (27%) 4 (5%) 1 (1%) 36 (42%) 34 (39%) 6 (7%) 3 (3%) 2 (2%) 69 (80%) 15 (17%) 11 (13%) 6 (7%) 9 (10%) 3 (3%) 2 (2%) 1 (1%)	**ESR/CRP** Tested in Elevated **Anemia** Tested in Present **Other deficiency** Tested in Vitamin D Vitamin B6 Vitamin A Vitamin B1 Vitamin B12 Vitamin B9 Folate Vitamin K	42 25(59%) 83 38(46%) 45 27 60%) 4 (9%) 4 (9%) 4 (9%) 2 (4%) 2 (4%) 1 (2%) 1 (2%)	**X-ray** Assessed in Frankel lines Wimberger ring Pelkan spur Trummerfeld zone Osteopenia Normal **CT** Assessed in Abnormal Normal **MRI** Assessed in Abnormal Normal	125 91(73%) 51(41%) 23(18%) 17(14%) 39(31%) 9 (7%) 6 5 (83%) 1 (17%) 36 35(97%) 1 (3%)	**Oncologic conditions** Leukemia Bone tumor Intracranial tumor **Infectious diseases** Osteomyelitis/SA Myositis Dental abscess Spondylodiscitis Meningoencephalitis **Inflammatory diseases** CNO JIA **Others** Guillain-Barré Spinal cord disease Fracture	**31** 25(52%) 5 (10%) 1 (2%) 21 14(29%) 3 (6%) 2 (4%) 1 (2%) 1 (2%) **11** 8 (17%) 3 (6%) **5** 2 (4%) 2 (4%) 1 (2%)

## Supplementary Materials

The following are available online at https://www.mdpi.com/article/10.3390/nu14030684/s1, Table S1: Epidemiological and clinical data, laboratory and radiological findings, treatment and outcome of the selected papers in the narrative review.
