# Supplementary material for: A Narrative Review on Pediatric Scurvy: The Last Twenty Years
Source: Nutrients. 2022 Feb 6;14(3):684. doi: 10.3390/nu14030684 (PMC8840722; doi:10.3390/nu14030684)
Supplement: Supplementary file 1 [file nutrients-14-00684-s001.zip › nutrients-1567489-supplementary.pdf]

Supplementary Materials

**Table S1.** Epidemiological and clinical data, laboratory and radiological findings, treatment and outcome of the selected papers in the narrative review									
**Author Year**	**# cases**	**Median age (months)**	**Male (%)**	**Comorbidity (%)**	**Clinical manifestations (n, %)**	**Laboratory data (n, %)**	**Radiological findings**	**Therapy Vitamin C**	**Outcome Timing (%)**
Ceglie et al 2018 [18]	3	29	100%	0	Musculoskeletal: 3 (100%)<br>-leg pain/refusal to walk 2<br>-limping, arthritis 1<br>Cutaneous: 1 (33%)<br>-petechiae 1<br>Mucous: 2 (67%)<br>-gingival swelling 2<br>Malaise/anorexia: 2 (67%)<br>Poor nutritional status 2 (67%)	Anemia: 2 (67%)<br>Vitamin D Δ: 2 (67%)<br>Folate Δ: 1 (33%)<br>Hypoalbuminemia: 1 (33%)	RX<br>osteopenia, Frankel's lines, Trummerfeld zone 1<br>widening of the anterior rib ends at costochondral junctions, Frankel's lines 1<br>MRI<br>inflammatory alterations of the soft tissue and cortical bone 1	IV followed by oral 2 (67%)<br>Oral 1 (33%)	Recovery<br>1 month<br>(100%)
Chalouhi et al 2020 [45]	3	36	33%	0	Musculoskeletal: 3 (100%)<br>-leg pain/refusal to walk 3<br>Cutaneous: 1 (33%<br>-inflammatory hair follicles 1<br>Mucous: 2 (67%)<br>-hyperemic gums 1<br>-conjunctival bleeding 1<br>Irritability: 3 (100%)<br>Malaise/anorexia: 2 (67%)<br>Fever: 1 (33%)<br>Poor nutritional status 1(33%)	-	RX<br>osteopenia, Frankel's lines, Trummerfeld zone 1<br>vertebral osteopenia 1<br>normal 1<br>MRI<br>metaphyseal symmetric bone-marrow signal changes of long bones 1<br>multifocal symmetric bone-marrow signal anomalies at the metaphysis, sub-periosteal collections, periosteal reaction 1	Oral: 3 (100%)	Recovery (100%)
De Ioris et al 2016 [5]	4	-	-	100%<br>cerebral palsy 3<br>Rett's S. 1	Musculoskeletal: 3 (75%)<br>-leg pain 3<br>-arthritis 2<br>Cutaneous: 3 (75%)<br>-petechiae, ecchymosis 3	Anemia: 2 (50%)	-	Oral: 4 (100%)	Recovery<br>3 months<br>(100%)

					Mucous: 3 (75%)<br>-gingival swelling 3<br>-gingival bleeding 2<br>Irritability: 3 (75%)				
Fortenberry et al, 2020<br>[49]	5	120	100%	100%<br>autism 5<br>cerebral palsy 1<br>ADHD 1	Musculoskeletal: 5 (100%)<br>-leg pain/refusal to walk 3<br>-limping 2<br>-arthritis 1<br>-leg edema<br>-back pain<br>Cutaneous: 4 (80%)<br>-petechiae 4<br>-ecchymosis 2<br>-perifollicular hemorrhages 1<br>Mucous: 4 (80%)<br>-gingival swelling 4<br>-gingival bleeding 4<br>-epistaxis 1<br>Fever: 3 (60%)<br>Hyperreactivity: 1 (20%)<br>Anorexia: 1 (20%)	Anemia: 2 (40%)<br><br>Vitamin D Δ: 4 (80%)<br>Vitamin B12 Δ: 1(20%)<br>Vitamin B1 Δ: 1 (20%)	RX<br>osteopenia 2<br>normal 1<br>MRI<br>enhancement of the margins of sacrum and ilium, the posterior iliac bones, and the pelvis apophyses 1	Oral 3 (60%)<br><br>IV followed by oral 1<br>rectal 1	Lost at FU 3 (60%)<br><br>Improvement at discharge 2 (40%)
Golriz et al 2016<br>[3]	32	132	59%	100%<br>TIRO 20<br>BMTR 3<br>Autism 3<br>DD 1<br>fructose intol. 1<br>neglect 1	-	-	RX<br>Frankel line 3; Trummerfeld zone 3<br>MRI<br>increased T2 signal in the bone marrow of metaphyses, periosteal reaction and edema at femurs and tibias 3<br>soft tissue edema 2	-	-
Gulko et al 2014<br>[47]	4	48	100%	100%<br>autism 2<br>Asperger's 1<br>DD 1	Musculoskeletal: 4 (100%)<br>-leg pain/refusal to walk 4<br>-arthritis 1<br>Cutaneous: 2 (50%)<br>-petechiae 1<br>-ecchymosis 1	High inflammatory markers: 2 (50%)<br>high CRP 1<br>high ESR 1<br><br>Vitamin D Δ: 1 (25%)	RX<br>normal 1<br>physeal widening with Frankel's lines 3<br>small metaphyseal fracture 1<br>MRI<br>multifocal symmetric signal abnormality	Oral: 4 (100%)	Improvement<br>4 (100%)

					-hyperkeratosis 1<br>Mucous: 2 (50%)<br>-gingival swelling 1<br>-gingival bleeding 1	Vitamin A Δ: 1 (25%)	at metaphyses with bone marrow enhancement 1<br>multifocal symmetric low signal on T1 and high signal on T2 at metaphyses; periosteal reaction; bone marrow enhancement 1<br>low signal on T1 and high signal on T2 at metaphyses 1<br>low signal on T1 and high signal on T2 at metaphyses; periosteal reaction; bone marrow<br>enhancement 1		
Kitcharoensakkul et al 2014 [50]	3	5	67%	67%<br>DD 2<br>autism 1	Musculoskeletal: 3 (100%)<br>-leg pain/refusal to walk 3<br>Cutaneous: 1 (33%)<br>-hyperkeratosis 1<br>Mucous: 2 (67%)<br>-gingival swelling 2<br>-gingival bleeding 2<br>Poor nutritional status: 1 (33%)	High inflammatory markers: 1 (33%)<br>ESR 30 mm/h<br><br>Vitamin D Δ: 2 (67%)<br>Vitamin A Δ: 1 (33%)	RX<br>Diffuse osteopenia, metaphyseal sclerotic lines 1<br>Frankel's lines in distal femur and proximal tibia 1<br>osteopenia and growth arrest lines in distal femurs and proximal tibias 1<br>MRI<br>Normal 1<br>Bone marrow edema with bilateral enhancement of distal femoral and proximal tibial metaphyses, edema of the periosteum and muscle 1	Oral: 3 (100%)	Lost at FU: 1 (33%)<br><br>Improvement 2 (67%)
Ma et al 2015 [4]	7	96	100%	100%<br>DD 7	Musculoskeletal: 7 (100%)<br>-leg pain/refusal to walk 7<br>-upper limb pain 1<br>Cutaneous: 5 (71%)<br>-petechiae, ecchymosis 5<br>-perifollicular rash 1<br>Mucous: 5 (71%)<br>-gingival swelling 5<br>-gingival bleeding 5<br>-epistaxis 1	High inflammatory markers: 2 (29%)<br>ESR 44-30 mm/h<br>CRP unspecified-1.8 mg/dl<br><br>Vitamin D Δ: 5 (71%)<br>Vitamin B1 Δ: 2 (29%)	RX<br>Widening and irregularity of bilateral distal femoral and proximal tibial physis 1<br>Normal 1<br>upon review, widening of the physis and Frankel's line 1<br>non-specified alterations 4<br>MRI<br>Bone marrow signal abnormalities at distal femur 1	Oral: 7 (100%)	Recovery 1-2 months (100%)

					Malaise: 2 (29%)<br>Poor nutritional status: 1 (14%)	Vitamin B6 Δ: 2 (29%)<br>Vitamin A Δ: 1 (14%)	Epiphyseal signal and diffuse infiltrative marrow process in femur and tibia metaphyses, edema and enhancement in soft tissues 1<br>Symmetric marrow edema and metaphyseal enhancement at femurs, tibias and fibulas; synovial enhancement and myositis 1<br>Bone marrow T2 hyperintensity and enhancement of the femoral metaphyseal, pelvis 1<br>Non-specified alterations 1		
Pan et al 2021 [48]	9	72	78%	67%<br>autism 4<br>hydrocephalus 1<br>tuberous sclerosis 1	Musculoskeletal: 7 (78%)<br>-leg pain/refusal to walk 7<br>-limping 7<br>-limb edema 4<br>Cutaneous: 6 (67%)<br>-petechiae, ecchymosis 6<br>-perifollicular hemorrhage 2<br>Mucous: 4 (44%)<br>-gingival bleeding 3<br>-epistaxis 1<br>Malaise: 2 (22%)<br>Fever: 1 (11%)<br>PH: 1 (11%)<br>Poor nutritional status: 1(14%)	high inflammatory markers: 5 (55%), median ESR 22 mm/h<br>median CRP 1.1 mg/dl<br><br>Anemia: 5 (55%)<br><br>Vitamin D Δ: 5 (55%)<br>Vitamin B6 Δ: 2 (22%)<br>Vitamin B9 Δ: 2 (22%)<br>Vitamin B1 Δ: 1 (11%)<br>Vitamin A Δ: 1 (11%)<br>Vitamin K Δ: 1 (11%)	RX<br>Normal 2<br>Frankel's lines 6, Osteopenia 2, Wimberger ring 2, Trummerfeld zone 4, subperiosteal hemorrhage 2, Pelkan spur 1<br>MRI<br>increased signal and enhancement of the femoral, tibial, fibular metaphysis with bone marrow edema adjacent to the greater trochanter apophysis, osteitis of the femur 3	Oral: 6 (67%)<br><br>IV followed by oral: 3 (33%)	Recovery 1-11 months (100%)
Ratageri et al 2005 [44]	3	12	100%	100%<br>infantile tremor syndrome	Musculoskeletal: 2 (67%)<br>-leg pain 2<br>Poor nutritional status: 3(100%)	Anemia: 3 (100%)	RX<br>Frankel's lines 3	Oral: 3 (100%)	Improvement (100%)

Ratanachu-E et al 2003 [53]	28	29	60%	7% cerebral palsy 2	Musculoskeletal: 25 (100%)<br>-leg pain/refusal to walk 25<br>-limping 24<br>-arthritis 13<br>Cutaneous: 1 (4%)<br>-Petechiae 1<br>Mucous: 12 (43%)<br>-gingival bleeding 12<br>Fever: 5 (21%)<br>PH 2 (7%)<br>Poor nutritional status 12 (43%)	Anemia: 19 (68%)	RX<br>Frenkel's lines 28, osteopenia 22, Pelkan spur 9, Winberger ring 8, subepiphyseal infarction 4, epiphyseal separation 2	Oral: 28 (100%)	recovery 1-4 weeks (100%)
Rubino et al 2020 [46]	4	42	100%	50% cerebral palsy 1 autism 1	Musculoskeletal: 4 (100%)<br>-leg pain/refusal to walk 4<br>-arthritis 2<br>Cutaneous: 3 (75%)<br>-petechiae, ecchymosis 3<br>Mucous: 4 (100%)<br>-gingival bleeding 3<br>-gingival swelling 1<br>Irritability: 3 (75%)<br>Poor nutritional status 1 (25%)	High inflammatory markers: 4 (100%)<br>median ESR 76 mm/h<br>median CRP 2.3 mg/dl<br><br>Anemia: 2 (50%)<br><br>Vitamin D Δ: 3 (75%)	RX<br>Frankel's lines 3, Trummerfeld zone 3, osteopenia 2, Pelkan spur 1<br>MRI<br>multifocal metaphyseal alterations 4	Oral: 4 (100%)	Recovery 2- 4 weeks
Singh et al 2015 [75]	48	-	-	100% malnutrition 48	-	-	RX<br>typical scurvy alterations 48 (Wimberger ring 41, Frankel's line 41, pencil-thin cortex 36, corner sign 21, Pelkan spur 12, Trummerfeld zone 2	-	-
Swed-Tobia et al, 2019 [52]	3	7	67%	100% Autism 3	Musculoskeletal: 3 (100%)<br>-leg pain/refusal to walk 3<br>-knee contracture 1<br>Cutaneous: 3 (67%)<br>-petechiae, ecchymosis 3	High inflammatory markers: 2 (67%)<br><br>Anemia: 2 (67%)	RX<br>normal 3<br>MRI<br>Intramedullary edema of vertebrae sacrum, sacroiliac joints, acetabulum, pubis, femurs 1	IV followed by oral: 3 (100%)	Improvement few days (100%)

					Mucous: 3 (67%)<br>-gingival bleeding 3		Intramedullary edema of right femur and pubis with a periosteal reaction, muscle reaction 1		

ADHD: Attention Deficit Hyperactivity Disorder; BMTR: bone marrow transplant recipient; CRP: C reactive protein; DD: developmental delay; ESR: erythrocyte sedimentation rate; FU: follow-up; IV intravenous; MRI: magnetic resonance imaging; ND: neurological diseases; PH pulmonary hypertension; RX: radiograph; TIRO: transfusion iron related overload; Δ : deficiency
